# Trichostatin A Triggers an Embryogenic Transition in Arabidopsis Explants via an Auxin-Related Pathway

**DOI:** 10.3389/fpls.2018.01353

**Published:** 2018-09-13

**Authors:** Barbara Wójcikowska, Malwina Botor, Joanna Morończyk, Anna Maria Wójcik, Tomasz Nodzyński, Jagna Karcz, Małgorzata D. Gaj

**Affiliations:** ^1^Department of Genetics, University of Silesia in Katowice Katowice, Poland; ^2^Department of Molecular Biology and Genetics, Medical University of Silesia Katowice, Poland; ^3^Mendel Centre for Genomics and Proteomics of Plants Systems, CEITEC MU – Central European Institute of Technology, Masaryk University Brno, Czechia; ^4^Scanning Electron Microscopy Laboratory, University of Silesia in Katowice Katowice, Poland

**Keywords:** *Arabidopsis thaliana*, auxin, epigenetics, histone acetylation, *in vitro* culture, somatic embryogenesis, transcription factors, trichostatin A

## Abstract

Auxin is an important regulator of plant ontogenies including embryo development and the exogenous application of this phytohormone has been found to be necessary for the induction of the embryogenic response in plant explants that have been cultured *in vitro*. However, in the present study, we show that treatment of Arabidopsis explants with trichostatin A (TSA), which is a chemical inhibitor of histone deacetylases, induces somatic embryogenesis (SE) without the exogenous application of auxin. We found that the TSA-treated explants generated somatic embryos that developed efficiently on the adaxial side of the cotyledons, which are the parts of an explant that are involved in auxin-induced SE. A substantial reduction in the activity of histone deacetylase (HDAC) was observed in the TSA-treated explants, thus confirming a histone acetylation-related mechanism of the TSA-promoted embryogenic response. Unexpectedly, the embryogenic effect of TSA was lower on the auxin-supplemented media and this finding further suggests an auxin-related mechanism of TSA-induced SE. Congruently, we found a significantly increased content of indolic compounds, which is indicative of IAA and an enhanced DR5::GUS signal in the TSA-treated explants. In line with these results, two of the *YUCCA* genes (*YUC1* and *YUC10*), which are involved in auxin biosynthesis, were found to be distinctly up-regulated during TSA-induced SE and their expression was colocalised with the explant sites that are involved in SE. Beside auxin, ROS were extensively accumulated in response to TSA, thereby indicating that a stress-response is involved in TSA-triggered SE. Relevantly, we showed that the genes encoding the transcription factors (TFs) that have a regulatory function in auxin biosynthesis including *LEC1, LEC2, BBM*, and stress responses (*MYB118*) were highly up-regulated in the TSA-treated explants. Collectively, the results provide several pieces of evidence about the similarities between the molecular pathways of SE induction that are triggered by TSA and 2,4-D that involve the activation of the auxin-responsive *TF* genes that have a regulatory function in auxin biosynthesis and stress responses. The study suggests the involvement of histone acetylation in the auxin-mediated release of the embryogenic program of development in the somatic cells of Arabidopsis.

## Introduction

Epigenetic modifications of DNA and histones are believed to play a pivotal role in controlling the development processes in animals and plants (reviewed by Feng et al., 2010; Lauria and Rossi, 2011). Among the epigenetic processes, the methylation of DNA and histone acetylation have been the most intensively studied regulatory mechanisms that control gene expression (Eberharter and Becker, 2002; Zilberman et al., 2007; Stricker et al., 2017).

The acetylation of lysine residues on the N-terminal tails of histones results in the removal of their positive charge, which alters the histone-histone and histone-DNA interaction and changes the accessibility of DNA to the chromatin-binding proteins (Turner, 2000). Hence, the acetylation of histones is believed to be associated with the open chromatin state and the activation of gene transcription, whereas the hypoacetylation of histones is characteristic for heterochromatin and gene silencing (Feng and Michaels, 2015).

Two families of antagonistically acting enzymes, histone acetyltransferases (HATs) and histone deacetylases (HDACs), are responsible for the dynamic changes in the state of histone acetylation. HATs use acetyl-coenzyme A (acetyl-CoA) to catalyze the addition of the acetyl group onto the ε-amino group of the lysine side chains, while HDACs remove this histone mark (Steunou et al., 2013). In Arabidopsis, the HAT proteins are divided into four types (GNAT, MYST, p300/CBP, and TAF1/TAF_II_250) based on their primary homology with yeast and the mammalian enzymes. The Arabidopsis HDAC family consists of 18 members that belong to three subfamilies, RPD3/HDA1, SIR2, and the plant-specific HD2 family (Pandey et al., 2002; Alinsug et al., 2009). The interplay between the HAT and HDAC enzymes contributes to the control of many biological processes including embryo development, seed dormancy and germination, morphogenesis, light signaling and flowering of Arabidopsis (reviewed by Boycheva et al., 2014; Wang et al., 2014). Moreover, histone acetylation has been postulated to regulate the transcription of the genes that control the stress responses (Liu et al., 2014) and plant-to-plant interactions (Venturelli et al., 2015).

Moreover, the role of the epigenetic processes in somatic cell dedifferentiation and plant regeneration from explants that have been cultured *in vitro* has also been postulated (Us-Camas et al., 2014). Changes in both DNA methylation and histone modifications have been reported during the cellular differentiation and embryogenic transition that was induced in the tissue of various plants that have been cultured *in vitro* (LoSchiavo et al., 1989; Santos and Fevereiro, 2002; Leljak-Levanić et al., 2004; He et al., 2011; Nic-Can et al., 2013; Ikeuchi et al., 2015; Mozgová et al., 2017). Congruent with the postulated involvement of histone acetylation in the embryogenic reprogramming of plant somatic cells, the genes encoding members of the HAT and HDAC families were found to be up-regulated during SE induction in Arabidopsis (Wickramasuriya and Dunwell, 2015). Moreover, the *hda9* and *hdt1* mutants of Arabidopsis showed a reduced callus formation capacity (Lee et al., 2016) and the *hac1* mutant exhibited a delayed initiation of shoot regeneration from the calli (Li et al., 2011). The chromatin decondensation and transcriptional activation of the genes that accompany the dedifferentiation of the protoplasts isolated from tobacco leaves were found to be associated with an increased level of acetylated lysine 9 and 14 in histone 3 (Williams et al., 2003). In *Pinus radiata*, immature needles that exhibited a high capacity for *in vitro* shoot organogenesis displayed a greater accumulation of histone 4 acetylation (H4Ac) compared to the mature needles, which showed a less efficient organogenic response (Valledor et al., 2010). Similarly, an increased level of H3 and H4 acetylation (H3Ac and H4Ac), which is associated with the expression of the histone acetyltransferase genes, was demonstrated during the microspore embryogenesis of *Brassica napus* (Rodríguez-Sanz et al., 2014).

Together, these reports confirm that the acetylation status of histones, primarily H3 and H4, seems to control the developmental programs that are induced *in vitro* in somatic plant cells. However, knowledge about the role of specific histone marks including the acetylation of specific lysine residues in the molecular mechanism that controls the developmental plasticity in somatic plant cells remains limited.

Among the various experimental approaches that can be used to modify histone acetylation the use of TSA, which is an antifungal antibiotic that is isolated from *Streptomyces hygroscopicus* that inhibits HDAC activity, has been recommended (Tsuji et al., 1976; Yoshida et al., 1995). TSA targets the RPD3/HDA1 and HD2-type subfamilies of the HDACs enzymes (Brosch et al., 1996; Jung et al., 1997) and the direct interaction of TSA with the active zinc site of RPD3/HDA1 HDACs was also shown (Finnin et al., 1999). In plants, the TSA-mediated inhibition of HDACs resulted in chromatin conformational changes that enhance gene expression due to histone hyperacetylation (Görisch et al., 2005). Relevantly, TSA treatment has been found to cause an increase of the H3K9/K14Ac and H4K5Ac epigenetic marks in the TSA-treated seedlings of Arabidopsis (Venturelli et al., 2015; Mengel et al., 2017).

In mammals, TSA has been widely used to improve the nuclear reprogramming of somatic cells for embryo cloning (Beigh et al., 2017; Miyamoto et al., 2017). Moreover, the TSA-induced inhibition of tumor growth and the apoptosis of cancer cells indicates the potential application of this drug in epigenetic therapy against cancer (Damaskos et al., 2017). While the potential of TSA to induce somatic cell reprogramming in plants was demonstrated, in contrast to mammals, the number of reports on TSA-treated plant material remains limited. TSA promoted the initiation of embryogenic tissue in Arabidopsis seedlings (Tanaka et al., 2008) and explants of conifers, *Picea abies* and *Pinus sylvestris* cultured *in vitro* (Uddenberg et al., 2011; Abrahamsson et al., 2017). In addition, the beneficial effects of TSA on cultures of the microspores of *Triticum aestivum* (Jiang et al., 2017) and *B. napus* (Li et al., 2014) have been reported. However, the genetic mechanism of the TSA-promoted embryogenic response of somatic plant cells has not yet been identified.

The numerous *TF*s that are differentially expressed during SE induction in Arabidopsis are plausible targets of TSA (Gliwicka et al., 2013) including *LEAFY COTYLEDON1* (*LEC1*), *LEC2* (*LEAFY COTYLEDON2*), *AGL15* (*AGAMOUS LIKE15*), *BBM* (*BABY BOOM*), *WUS* (*WUSCHEL*), *MYB118*, and *EMK* (*EMBRYOMAKER*). These *TF*s positively regulate the vegetative-to-embryonic transition and their overexpression results in SE induction without auxin treatment (Lotan et al., 1998; Stone et al., 2001; Boutilier et al., 2002; Zuo et al., 2002; Harding et al., 2003; Wang et al., 2009; Tsuwamoto et al., 2010). The similarity of the genetic pathways that control the embryonic reprogramming of somatic cells in different plants seems to be plausible since overexpression of the Arabidopsis genes, *BBM*, *LEC2*, and *WUS*, was successfully used to improve the plant regeneration efficiency of *in vitro* recalcitrant crops (Solís-Ramos et al., 2009; Deng et al., 2009; Heidmann et al., 2011; Belide et al., 2013; Lowe et al., 2016).

However, controlling the developmental plasticity of plant somatic cells requires deciphering the epigenetic processes that regulate the expression of the SE-involved *TF*s that to date has been limited to the polycomb repressive complex 2 (PRC2). PRC2 regulates the phase transitions during plant development through the trimethylation of lysine 27 of histone 3 (H3K27me3) and its role in SE induction has been inferred given that the *clf swn* mutant in the genes encoding the catalytic subunits *curly leaf (CLF)* and *swinger (SWN)* is capable of somatic embryo development from the roots and shoots (Chanvivattana et al., 2004; Ikeuchi et al., 2015; Mozgová et al., 2017; Birnbaum and Roudier, 2017). Among the PRC2-controlled genes, *LEC2*, which has an essential function in SE induction, was identified (Gaj et al., 2005; Ikeuchi et al., 2015).

To gain insights into the role of histone acetylation in the embryogenic transition that is induced in somatic plant cells, we analyzed the developmental effects of TSA in the IZEs of Arabidopsis explants that were cultured *in vitro*. We found that in the absence of auxin treatment, TSA efficiently promoted somatic embryo development and that this process was associated with the extensive up-regulation of the *TF* genes that play a crucial role in SE induction. Among the TSA-activated genes were the *YUC* genes that are involved in auxin biosynthesis and the up-regulation of *YUC1* and *YUC10* was associated with a significant accumulation of auxin in the TSA-treated explants. Collectively, the results indicate an auxin-related mechanism of TSA-promoted SE induction.

## Materials and Methods

### Plant Material

The Columbia (Col-0) genotype of *Arabidopsis thaliana* (L.) Heynh and transgenic plants with the reporter construct in the Col-0 background were used. The Col-0 seeds were supplied by NASC (The Nottingham Arabidopsis Stock Centre). The seeds of the DR5::GUS line and the *YUC* (*pYUC1-GFP*, *pYUC10-GFP*) reporter lines were kindly provided by Jane Murfett (Division of Biological Sciences, University of Missouri, Columbia, MO, United States) and Helene Robert Boisivon (CEITEC MU-Central European Institute of Technology, Masaryk University, Mendel Centre for Genomics and Proteomics of Plants Systems, Brno, Czechia), respectively.

### *In vitro* Cultures of the Explants

IZEs at the cotyledonary stage of development were used as the explants for the *in vitro* cultures. In one experiment, cotyledons that had been cut off from freshly isolated IZEs were cultured. The explants were sterilized and cultured following the standard protocol for SE (Gaj, 2001) and shoot organogenesis (ORG) (Kraut et al., 2011) induction. In each culture combination, ten explants were cultured in one Petri dish (Ø 35 mm for SE or 60 mm for ORG) and thirty explants in three replicates were analyzed.

### SE Induction

The basal E0 medium contained 3.2 g L^-1^ of B5 (Gamborg et al., 1968) micro- and macro-elements (Duchefa Biochemie; #G0210), 20 g L^-1^ sucrose and 8 g L^-1^ agar, pH 5.8. The standard medium for SE induction (EA) contained 5.0 μM of 2,4-D. Other auxin media, which were supplemented with 0.5; 2.5; 3.5 μM of 2,4-D, were also used. In one experiment, 2,4-D was replaced with IAA (10; 30; 50 μM) and NAA (5; 10; 20 μM). In addition, an E0 medium that supplemented with TSA (Sigma Aldrich; #T1952) at concentrations of 0.1; 0.5 and 1.0 μM was used.

### Shoot Organogenesis

The IZEs were incubated for 7 days in a liquid CIM, then the cotyledons were cut off and cultured on solid shoot induction media (SIM-C) according to Kraut et al. (2011). The CIM medium contained a basal composition of a B5 medium (Gamborg et al., 1968), 0.5 g L^-1^ MES, 20 g L^-1^ glucose, 2.2 μM of 2,4-D and 0.2 μM of kinetin. The SIM-C medium contained the micro-elements of MS (Murashige and Skoog, 1962), macro-salts and vitamins of a B5 medium (Gamborg et al., 1968) and was supplemented with 30 g L^-1^ sucrose, 0.5 μM of NAA (1-naphthaleneacetic acid) and 4.4 μM of BAP (6-benzylaminopurine).

### Evaluation of the Embryogenic Capacity

The explant capacity for SE was evaluated in 21-day-old cultures and two parameters were calculated – SE efficiency (the percentage of explants that formed somatic embryos) and SE productivity (the average number of somatic embryos produced per explant). All of the culture combinations were evaluated in three replicates and at least 30 explants (ten explants/Petri dish) were analyzed per one replicate.

### Conversion of Embryo-Like Structures Into Plants

The capacity of the embryo-like structures to develop into plants (so-called conversion rate) was analyzed as was previously described (Nowak et al., 2012). The average conversion rate was based on three independent experiments and at least 100 of the embryo-like structures per replicate were evaluated.

### Plant Growth and *in vitro* Culture Conditions

The seed-derived plants were grown in Jiffy-7 (Jiffy, Norway) pots at 22°C under a 16 h photoperiod of 100 μM m^-2^ s^-1^ white, fluorescent light. The plant materials that were grown in sterile conditions were kept at 23°C under a 16/8 h photoperiod of 40 μM m^-2^ s^-1^ white, fluorescent light.

### Lipid Staining

Sudan red 7B staining, which is indicative of neutral lipids (Brundrett et al., 1991), was used. The explants were stained overnight in a filtered Fat Red (Sigma Aldrich) solution (0.05%), rinsed with distillated water and examined under a stereomicroscope.

### Superoxide Anion Detection

In order to detect ROS, nitroblue tetrazolium chloride (NBT), which detects superoxide anion (O_2_^.-^), was applied (Rossetti and Bonatti, 2001). The explants were stained in a solution of 2 mM NBT (Invitrogen) in a 20 mM sodium phosphate buffer (pH 6.1) for 30 min and then they were washed in distilled water and examined under a stereomicroscope.

### GUS Detection

Explants of the DR5::GUS line cultured on the E0 and ET (1.0 μM TSA) media for 0, 5, 10, 15, 21, and 28 days were sampled and incubated in a GUS assay solution at 37°C for 12 h (Jefferson et al., 1987). Pigments from tissue were removed with 95% ethanol.

### Processing of Samples for Scanning Electron Microscopy (SEM)

Explants from different time points of the SE cultures were collected and fixed in 4% PFA in PBS with 0.1% Tween for 3 h at room temperature in a vacuum. Next, the samples were rinsed for 30 min in methanol and washed 3 × 5 min in 100% ethanol. After fixation, the samples were washed in PBS and dehydrated in an ethanol series (30, 50, 70, 80, 90, 95, and 100%) for 10 min each, followed by replacing the ethanol with acetone. The dehydrated samples were dried with a CPD 2 critical-point drier (Pelco) using liquid carbon dioxide, mounted on aluminum stubs with double-sided adhesive carbon tape and sputter-coated with a 12.5 nm (0, 5, 10, and 15 days) or 20 nm (21 and 28 days) film of gold in anSC-6 sputter coater (Pelco). After processing, the samples were imaged using a Hitachi SU 8010 UHR FESEM field emission scanning electron microscope (Hitachi High-Technologies Corporation, Tokyo, Japan) at 5 kV accelerating voltage with a secondary electron detector (ESD).

### Microscope Analysis

A Zeiss Stemi 2000-C microscope was used to analyze the lipids, ROS accumulation and GUS signal, the images were saved as jpg files using an Axi-Vision Camera. Live-cell microscopy was performed on a Zeiss 700 confocal laser scanning microscope using a 488 nm emission filter to detect the GFP signal.

### Assessment of the Content of Indolic-Compounds

A colourimetric technique that permitted the detection of indolic compounds including IAA was used (Bric et al., 1991). The IZE explants cultured on the E0 and ET media for 5, 10, 15, 21, and 28 days were analyzed. The procedure was performed as was previously described (Wójcikowska et al., 2013). Accordingly, fresh tissue was transferred to mortars containing 2 mL of 10 × PBS immediately after it was harvested. The material was homogenized and the solution was centrifuged (25 min; 18,000 × *g*). Then, 2 mL of supernatant was mixed with 100 μL of 10 mM orthophosphoric acid and 4 mL of Salkowski’s reagent. The pink absorbance that developed after a 30 min incubation at room temperature was read at 530 nm. The IAA concentration was determined with the calibration curve of pure IAA as the standard following linear regression analysis. Each analysis was carried out in three biological replicates.

### Nuclear Extract Isolation

Following the protocol of Gendrel et al. (2005) with the minor modifications of Buszewicz et al. (2016), the nuclear proteins were isolated from the explants cultured on the EA, E0, and ET media for 0, 5, 10, and 15 days and 0.04–0.2 g of fresh tissue per sample was used. Protein concentration was estimated using the Bradford assay and absorbance was measured by Tecan Infinite M200 in Bio-one Cellstar 96-well plate (Greiner) with 595 nm wavelength. Samples were stored at -80°C.

### HDAC/HAT Enzymes Activity

The activity of the HAT and HDAC enzymes was measured in a nuclear protein extract and 1.8–8.2 μg of the extract was used per sample. The colourimetric ELISA method and commercially available kits *–* Epigenase HDAC Activity/Inhibition Direct Assay Kit^1^ and EpiQuik HAT Activity/Inhibition Assay Kit^2^ (Epigentek) were used. The procedure followed the manufacturer’s protocols. The ratio of acetylated/deacetylated histones was colourimetrically measured by reading the absorbance in a Tecan Infinite M200 Microplate reader with 450 nm wavelength. As a control to the blank, reader wells without the antigen or primary antibody were used. The activity of the HDAC, and HAT enzymes was proportional to the OD intensity that was measured. Three biological and two technical replicates of each culture combination were analyzed in order to calculate the average enzyme activity.

### Isolation of RNAs and miRNAs

A RNAqueous Kit (AMBION) and mirVana^TM^ Kit (AMBION) was used to isolate the total RNAs and miRNAs, respectively, from the IZE explants induced on the different media for 0, 5, 10, 15, 21, and 28 days. Depending on the age of the culture from 250 (day 0) to 4 (day 28) explants were used to isolate the RNAs/miRNAs. The concentration and purity of the RNA/miRNA was assessed using a ND-1000 spectrophotometer (NanoDrop).

### Reverse Transcription, Stem-Loop Reverse Transcription, RT-PCR and RT-qPCR Analyses

In order to control any DNA contamination, the RNAs were treated with RQ1 RNase-free DNase I (Promega) according to the manufacturer’s instructions. The first-strand cDNA was produced in a 20 μL reaction volume using a RevertAid First Strand cDNA Synthesis Kit (Fermentas).

The product of the reverse transcription was diluted with water at a 1:1 ratio and 1 μL of this solution was used for the RT-PCR reactions. A LightCycler^®^ 480 (Roche) real-time detection system was used for the RT-qPCR reactions to analyze the relative level of mRNAs and miRNAs using the gene/miRNA-relevant primers (**Supplementary Table S1**) following Wójcikowska and Gaj (2017) and Speth and Laubinger (2014), respectively. The product of the reverse transcription was diluted with water at a 1:4 ratio and 2.5 μL of this solution was used for the RT-qPCR reactions. The relative RNA levels were calculated and normalized to the internal control of the *AT4G27090* (*TIN*) gene encoded 60S ribosomal protein (Thellin et al., 1999). The relative expression level was calculated using 2^-ΔΔCT^ where ΔΔ*C*_T_ represented Δ*C*_T_^reference condition^ – Δ*C*_T_^compared condition^. The plant tissues for the gene expression analysis were produced in three biological replicates and two technical replicates were analyzed.

### Statistical Analysis

The Student’s *t*-test (*P* < 0.05) or two-way ANOVA analysis (*P* < 0.05) followed by Duncan’s test (*P* < 0.05) was used to determine any values that were significantly different between the combinations that were being compared. The figures show the averages from the biological replicates with the standard error.

## Results

### TSA Promotes an Embryogenic Response in Explants That Are Cultured on an Auxin-Free Medium

**FIGURE 1:**
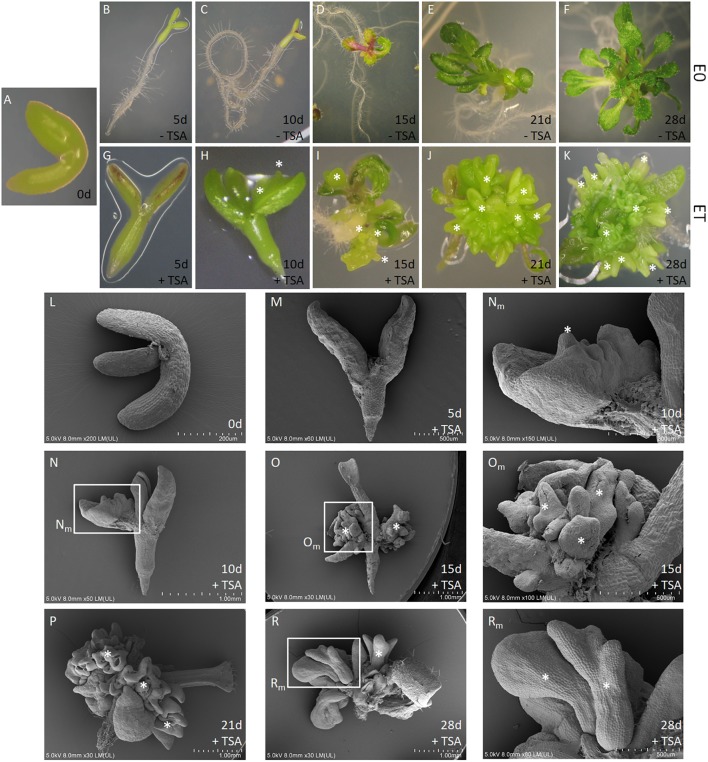
TSA promotes SE induction in *in vitro* cultured Arabidopsis explants. The IZE explants of Col-0 **(A)** developed into seedlings **(B–F)** and somatic embryos **(G–K)** on the control E0 and ET (E0 + 1.0 μM TSA) medium, respectively. SEM images **(L–R)** of the explants cultured on the ET medium. Explants at different time points are indicated as the day (d) of the culture: 0 **(A,L)**, 5 **(B,G,M)**, 10 **(C,H,N)**, 15 **(D,I,O)**, 21 **(E,J,P)**, and 28 **(F,K,R)**. Magnification views (**N_m_, O_m_, R_m_**) of the areas framed in **N, O, R.** An asterisk (^∗^) indicates somatic embryos.

**FIGURE 2:**
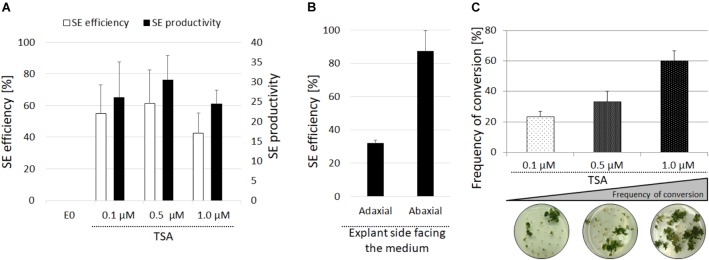
Evaluation of the embryogenic response of the Col-0 explants treated with TSA. SE efficiency and SE productivity were evaluated in the IZE explants of Col-0 cultured on the E0 medium with 0.1, 0.5, and 1.0 μM of TSA **(A)**. The SE efficiency of the isolated cotyledons placed on the adaxial and abaxial side on the E0 medium with 1.0 μM of TSA **(B)**. The conversion rate of the somatic embryos derived from the IZEs cultured on the medium with 0.1, 0.5, and 1.0 μM of TSA calculated as the frequency of the somatic embryos that developed into plants with roots (**C**) (*n* = 3; means ± SE).

In order to examine the role of histone acetylation in the epigenetic control of the embryogenic transition that was induced in the somatic plant cells, the effect of TSA was assessed in the IZE explants of Arabidopsis that were cultured *in vitro*. The IZE explants cultured on an auxin-free E0 medium developed into seedlings while supplementing the medium with TSA (0.1–1.0 μM) resulted in the formation of embryo-like structures (**Figures 1A–K**). SEM analysis indicated that the upper part of the explants including the cotyledons and SAM responded exclusively to the TSA treatment and that in the 10-day-old culture, the first protuberances started to emerge on the adaxial side of the cotyledons and somatic embryos were subsequently developed (**Figures 1L–R**). We found that 42–61% of the explants treated with 0.1–1.0 μM of TSA underwent SE induction and that an average of 27 of the embryo-like structures were produced per explant (**Figure 2A**). The cotyledons isolated from the IZEs also displayed an efficient SE induction and over 80% of the cotyledons located on the abaxial side on the medium that supplemented with 1.0 μM of TSA formed numerous somatic embryos on the upper, i.e., adaxial side (**Figure 2B**). In contrast, the cotyledons that had been placed on the medium in the opposite orientation displayed a significantly lower (up to 2.5-fold) embryogenic response. To summarize, the adaxial side of the IZE cotyledons responded to the TSA-induced embryogenic transition efficiently.

Upon their transfer onto the TSA-free medium, the TSA-induced embryo-like structures were capable of developing shoots with roots and the concentration of TSA used in the induction medium was positively correlated with the frequency of somatic embryos that regenerated complete plants. Accordingly, the somatic embryos induced on the ET medium with 1.0 μM TSA developed into plants with the highest efficiency of 60% (**Figure 2C**). This result implies that the majority of the embryo-like structures induced in the presence 1.0 μM of TSA represented complete somatic embryos with a functional shoot and root poles. Additional evidence of the embryonic identity of the TSA-induced structures was provided by staining with Sudan Red 7B, which marks the neutral lipids accumulate specifically in the embryonic tissue (Brundrett et al., 1991). We found that in contrast to the seedlings and adventitious shoots that had regenerated on the E0 and CIM/SIM media, respectively, the structures induced with TSA and auxin showed an intense red staining, thus indicating their embryonic character (**Supplementary Figure S1A**). In addition, the explants treated with TSA and auxin accumulated high level of ROS (**Supplementary Figure S1B**). To summarize, the results showed that TSA at a concentration of 1.0 μM efficiently triggered an embryogenic response in the IZE explants and that the majority of developing somatic embryos were bipolar embryos with the functional shoot and root poles.

### Decreased Activity of HDAC and HAT in the TSA-Treated Explants

**FIGURE 3:**
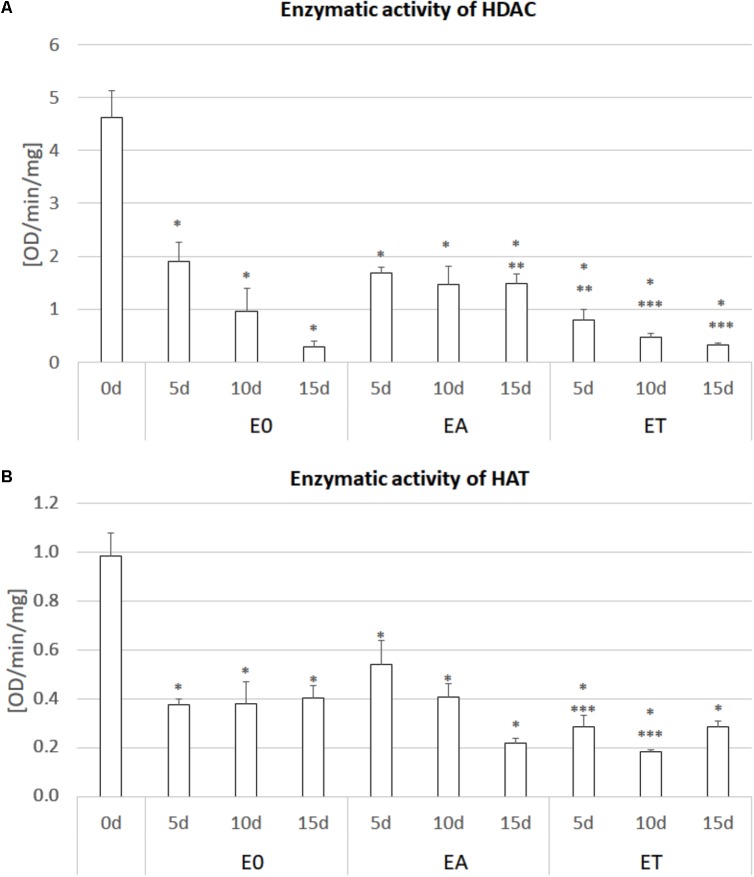
Enzymatic activity of HDAC **(A)** and HAT **(B)** in the IZE explants of the Col-0 cultured on the E0, EA (E0 + 5.0 μM 2,4-D) and ET (E0 + 1.0 μM TSA) media for 0, 5, 10, and 15 days. The enzyme activity was calculated as the OD/min/mg protein. A two-way ANOVA analysis (*P* < 0.05) followed by Duncan’s test (*P* < 0.05) was used to indicate values that were significantly different from: 0d (^∗^); the E0 culture at the same age (^∗∗^); the EA culture at the same age (^∗∗∗^) (*n* = 3; ± SE).

To verify the assumption that TSA promotes SE induction *via* a histone acetylation-related mechanism, we evaluated the activity of HDACs and HATs in the explants cultured on the ET medium with 1.0 μM of TSA (**Figure 3**). The analysis indicated a significant reduction of up to 93% of the HDAC activity in the explants cultured on the ET medium compared to the freshly isolated tissue (0 d). Surprisingly, the HDAC activity also decreased in the explants cultured on the TSA-free media, E0 and EA. Nevertheless, the HDAC activity was up to twofold lower in the explants cultured on the TSA-supplemented media.

In addition to HDACs, the activity of HATs was substantially reduced in the explants that have been cultured *in vitro* and the auxin- and TSA-treated cultures displayed the lowest activity of acetylases, which was up to fivefold lower than in the 0 d explants.

### Simultaneous Treatment of the Explants With TSA and Auxin Inhibited SE Induction

**FIGURE 4:**
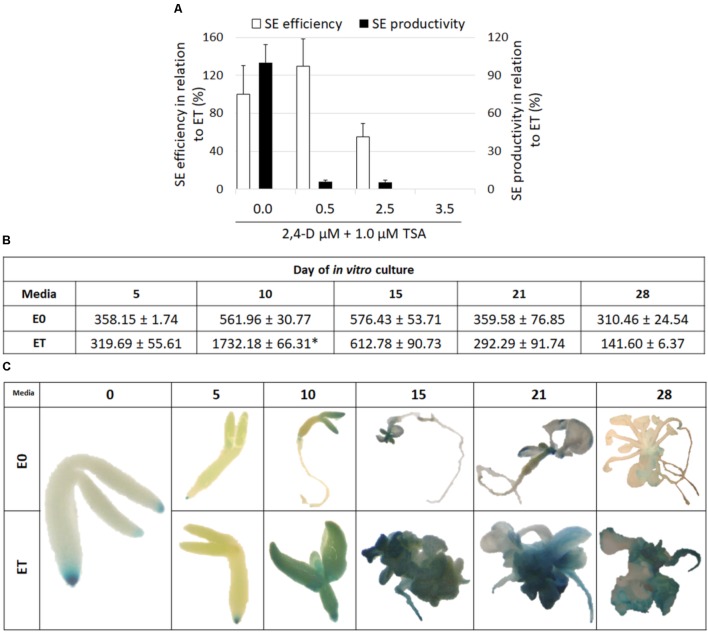
TSA-induced SE is associated with auxin accumulation. Auxin inhibits TSA-induced SE. SE efficiency and productivity was assessed in the IZE Col-0 explants cultured on the ET (E0 + 1.0 μM TSA) medium supplemented with different 2,4-D concentrations (0.0, 0.5, 2.5, and 3.5 μM) in relation to the ET medium that was set at 100% **(A)** (*n* = 3 ± SE; the Student’s *t*-test *P* < 0.05). The content of indolic compounds was analyzed in the explants cultured on the ET (E0 + 1.0 μM TSA) and E0 media; values significantly different from the E0-derived culture of the same age are indicated by an asterisk (^∗^) **(B**) (*n* = 3 ± SE; the Student’s *t*-test *P* < 0.05). Spatiotemporal localisation of the DR5::GUS signal, which was indicative of auxin accumulation in the explants cultured on the ET (E0 + 1.0 μM TSA) and E0 media **(C)**.

Because 2,4-D is commonly used to induce an embryogenic response in Arabidopsis, we addressed the question of the response of explants that had simultaneously been treated with 2,4-D and TSA. It was found that 2,4-D negatively affected the embryogenic response induced by TSA alone in a concentration-dependent manner. Total repression of the embryogenic response and the production of a non-embryogenic callus was observed in the explants induced on the ET medium supplemented with 3.5 μM of 2,4-D (**Figure 4A**). The inhibition of a TSA-induced embryogenic response was not specific to 2,4-D and it was also observed that other auxins, IAA and NAA, significantly inhibited SE induction on the ET medium (**Supplementary Figure S2**).

### Auxin Accumulation in the TSA-Induced Explants

Given the key role of auxin in SE induction, we decided to assess the auxin accumulation in the TSA-treated explants. To this end, the content of indolic compounds (ICs), which are indicative of IAA, was evaluated in the ET- vs. E0-induced explants and the analysis showed a significant, more than threefold increase of ICs in the 10 d culture treated with TSA (**Figure 4B**). Further evidence that TSA treatment results in an intensive auxin accumulation was provided by a strong DR5::GUS signal in the explants cultured on the ET medium (**Figure 4C**). In conclusion, we assume that the enhanced biosynthesis of IAA is associated with the TSA-promoted mechanism of SE induction.

### TSA Treatment Up-Regulates the *YUC1* and *YUC10* Genes That Are Involved in Auxin Biosynthesis

**FIGURE 5:**
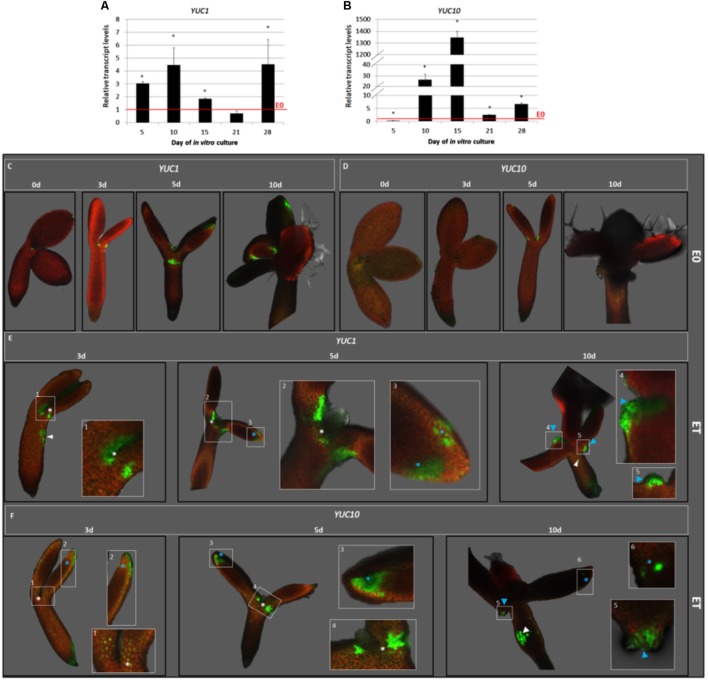
TSA up-regulated the *YUC1* and *YUC10* expression in the SE-involved explant parts. The RT-qPCR-monitored relative expression level of the *YUC1* **(A)** and *YUC10* **(B)** genes in the IZE explants of Col-0 cultured for 5, 10, 15, 21, and 28 days on the ET (E0 + 1.0 μM TSA) medium. The relative transcript level was normalized to the internal control (*At4g27090*) and calibrated to the E0 culture. Values that were significantly different to that on E0 at the same age are indicated with an asterisk (^∗^) (*n* = 3; means ± SE; the Student’s *t*-test *P* < 0.05). The GFP-monitored spatiotemporal expression pattern of the *YUC1* **(C,E)** and *YUC10* **(D,F)** genes in the IZE, Col-0 explants cultured for 0, 3, 5, and 10 days on a control E0 **(C,D)** and ET (E0 + 1.0 μM TSA) **(E,F)** media. GFP signals at different locations are indicated including the adaxial side of the cotyledons (blue asterisk); SAM-proximity (white asterisk); regenerating somatic embryos (blue arrowhead); hypocotyl (white arrowhead). White frames (1–6) indicate the magnified areas.

In order to identify the auxin biosynthesis genes that were activated in the embryogenic culture in response to TSA, the expression patterns of eleven member genes of the *YUCCA* family were analyzed in the explants cultured on the ET vs. E0 medium. A RT-PCR analysis indicated the expression of nine *YUC* genes (*YUC1*, *3*, *4*, *5*, *6*, *8*, *9*, *10*, and *11*) in the ET-induced culture (**Supplementary Figure S3**) and the transcription of *YUC1* and *YUC10* appeared to increase on the ET medium. The RT-qPCR analysis supported this assumption and we found that the *YUC1* and *YUC10* genes were substantially up-regulated in response to TSA and, in particular, the *YUC10* transcripts were intensively accumulated (over 1300-fold) (**Figures 5A,B**).

To confirm the contribution of the *YUC1* and *YUC10* genes to TSA-induced SE, we analyzed the spatiotemporal pattern of their expression in the *pYUC1:GFP* and *pYUC10:GFP* explants in the TSA- and E0-cultured explants (**Figures 5C–F**). The analysis revealed that in response to TSA, *YUC1*, and *YUC10* were expressed in the cotyledons and SAM-proximity, which are the explant regions that are involved in SE induction (**Figures 5E,F**). The GFP signal was scattered on the adaxial side of the cotyledons and was probably colocalised with the sites of the early SE induction (days 3–5). In the more advanced culture (day 10), the *YUC* expression had protuberances that emerged on the adaxial side of the cotyledons. Notably, we found that the expression of *YUC1* and *YUC10* was not exclusively limited to the SE-involved tissue and that the GFP signal was also observed in the hypocotyls that were incapable of SE.

### TSA-Treatment Results in the Extensive Up-Regulation of the SE-Involved *TF* Genes

**FIGURE 6:**
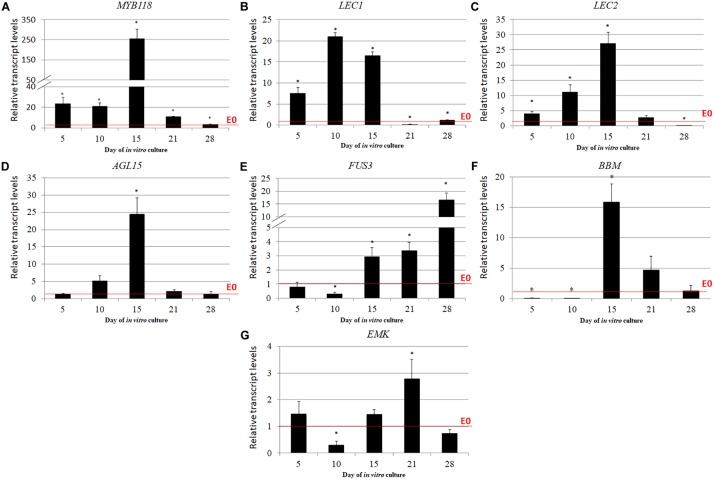
*TF* genes that have a regulatory role in SE induction were significantly up-regulated in response to the TSA treatment including *MYB118* **(A)**, *LEC1* **(B)**, *LEC2* **(C)**, *AGL15* **(D)**, *FUS3* **(E)**, *BBM* **(F)**, and *EMK* **(G)**. The IZE explants of Col-0 were cultured on an ET (E0 + 1.0 μM of TSA) medium and the tissue for the RT-qPCR analysis was sampled on days 5, 10, 15, 21, and 28 of the culture. The relative transcript level was normalized to the internal control (*At4g27090*) and calibrated to the culture on E0. Values that were significantly different to the culture on E0 at the same age are indicated by an asterisk (^∗^); (*n* = 3; means ± SE; the Student’s *t*-test *P* < 0.05).

The expression level of several *TF*s that have a reported contribution to SE induction including *LEC1*, *LEC2*, *FUS3*, *BBM*, *WUS*, *AGL15*, *EMK*, and *MYB118* were analyzed in the explants cultured on the ET vs. E0 medium. We observed that except for *WUS* whose transcripts were not detected in the explants cultured on both of the media that were used (**Supplementary Figure S4**), TSA treatment significantly increased the expression of analyzed *TF* genes over the level that was observed on the E0 medium (**Figures 6A–G**). *MYB118*, whose transcript level increased up to 255-fold in the 15 d culture induced by TSA, displayed the highest up-regulation (**Figure 6A**). The other *TF*s including *LEC1*, *LEC2*, *AGL15*, *FUS3*, and *BBM* were up-regulated at least 15-times more in response to TSA and for the majority of them, the maximal transcript accumulation was observed in the culture induced on the ET medium for 15 days (**Figures 6B–F**). Notably, the expression level of the *TF* genes in the TSA-induced culture substantially exceeded those that were observed in the auxin-induced explants and, in particular, the *LEC2* transcripts were highly accumulated and their level was more than 125-times higher in response to TSA- compared to the 2,4-D-treatment (**Supplementary Figure S5**).

**FIGURE 7:**
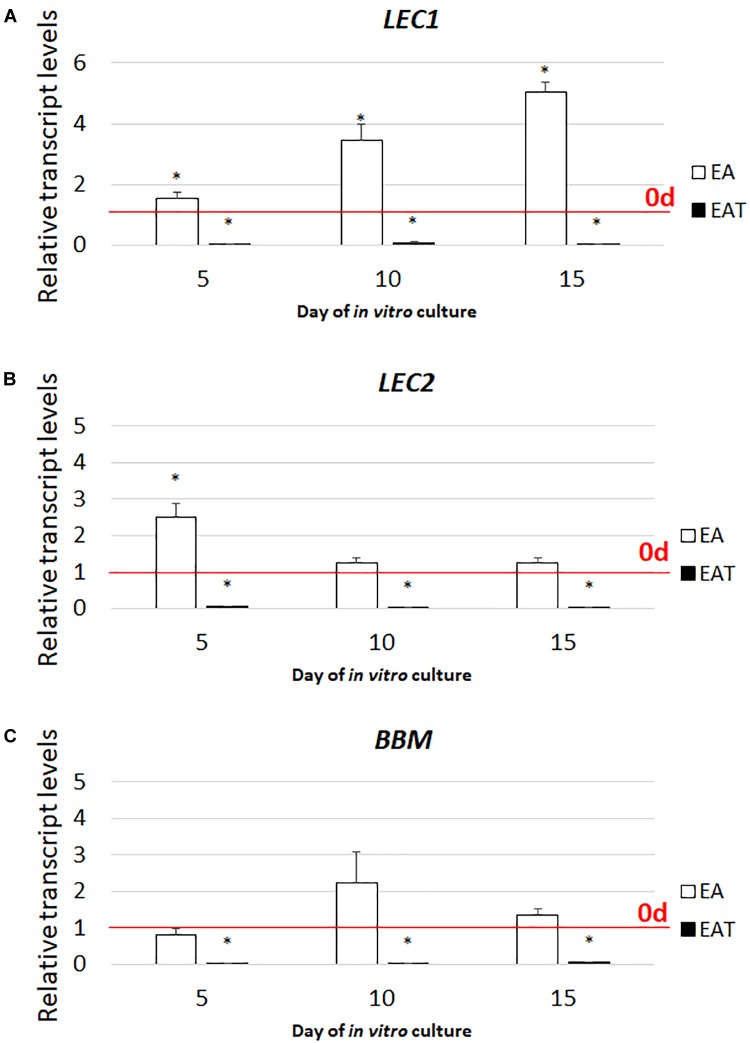
Combined treatment with TSA and 2,4-D represses the expression of the *LEC1* **(A)**, *LEC2* **(B)**, and *BBM* **(C)** genes. The IZE explants of Col-0 cultured on the EA (5.0 μM of 2,4-D) and EAT (1.0 μM of TSA + 5.0 μM of 2,4-D) media and the tissue for the RT-qPCR analyses was sampled on days 5, 10, 15 of the culture. Relative transcript level was normalized to the internal control (*At4g27090*) and calibrated to the 0 d. Values significantly different to that observed on 0 d is indicated by an asterisk (^∗^); (*n* = 3; means ± SE; the Student’s *t*-test *P* < 0.05).

Given that the simultaneous treatment of the explants with TSA and auxin inhibited the embryogenic response (**Figure 4A** and **Supplementary Figure S2**), we were curious about the expression level of the key SE-regulators in the explants cultured on the ET medium with 2,4-D. Therefore, *LEC1*, *LEC2*, and *BBM*, which control auxin biosynthesis were analyzed and a strong inhibition of their expression was observed in the culture induced on the ET medium supplemented with 5 μM of 2,4-D (**Figure 7**). This suggests that the inhibition of the embryogenic response that was observed on the ET medium with 2,4-D resulted from the reduced expression of the *TF* genes that have a key function in SE induction.

## Discussion

The reprogramming of differentiated cells toward an embryonic state, which requires the remodeling of chromatin in order to break the epigenetic barriers and histone acetylation in concert with the methylation of histones and DNA are believed to play central roles in this process reviewed in Fehér (2015) and Horstman et al. (2017a). In support of the function of histone acetylation in cellular reprogramming, the inhibitor of HDACs – TSA – has been shown to improve embryo cloning in mammals (Beigh et al., 2017; Miyamoto et al., 2017) and to promote somatic embryo development in Arabidopsis seedlings (Tanaka et al.; 2008). Given that the TSA treatment was observed to have a distinct impact on gene expression in animals and plants (Görisch et al., 2005; Chang and Pikaard, 2005; Inoue et al., 2015), the embryogenic response that is induced by TSA appears to result from the de-repression of the specific regulatory genes that control the embryogenic transition. To gain insight into the molecular mechanism of TSA-induced embryogenesis, we analyzed the effects of TSA in IZE of Arabidopsis cultured *in vitro* that provide a model system for studies on the molecular factors that govern the embryogenic response of somatic plant cells (Wójcikowska and Gaj, 2016).

### Similarity of TSA- and 2,4-D-Induced Embryogenic Response

We demonstrated that the TSA-treated IZE explants underwent SE induction in the absence of exogenous auxin (2,4-D), which is a standard SE-inducer in Arabidopsis and other plants (Horstman et al., 2017a). Alike 2,4-D-induced SE, the TSA-triggered embryogenic transition was induced rapidly with only sporadic callus production and exclusively the adaxial side of cotyledons responded to SE induction (Kurczyńska et al., 2007; Gaj, 2011). Moreover, we found that the TSA-triggered embryogenic response was highly efficient because up to threefold more somatic embryos were produced per explant in the TSA (present results) than in the 2,4-D-induced (Gaj, 2011) cultures. The cotyledons detached from the IZEs also displayed a capacity for TSA-induced SE, thereby confirming a high and autonomous embryogenic potential of the cotyledon tissue in Arabidopsis. Most importantly, we found a distinct difference in the TSA-responsiveness between the adaxial and abaxial sides of the cotyledons as somatic embryos were generated almost three times more frequently from the adaxial side of the cotyledons. We assumed that the specific expression pattern of genes in the adaxial tissue might account for the enhanced capacity of this tissue for SE induction. Relevantly, the SE-involved *PHB* (Wójcik et al., 2017) is active exclusively on the adaxial side of the cotyledons/leaves as a result of the miR165/166-mediated *PHB* repression in the abaxial region (Kidner and Martienssen, 2004; Grigg et al., 2009). In addition, the *YUCCA*s and *ARF*s genes, which play key roles in the auxin-induced embryogenic transition (Wójcikowska et al., 2013; Wójcik et al., 2017), have been found to be differently expressed in the adaxial and abaxial tissue (Garcia et al., 2006; Chitwood et al., 2009; Wang et al., 2011; Machida et al., 2015; Guan et al., 2017).

We found that TSA treatment with 1.0 μM was the most efficient in inducing the high frequency of bipolar somatic embryos that developed into shoots with roots, while a lower concentration (0.1 μM) resulted in incomplete somatic embryos. Similarly, 2,4-D-treatment affected the frequency of bipolar somatic embryos in a concentration-dependent manner (Raghavan, 2004; Nowak et al., 2012). Given that a specific auxin treatment was required in order to activate the root-specific *TF*s (*PLETHORA1*, *2* and *WUSCHEL-RELATED HOMEOBOX5*) in the somatic embryos (Mozgová et al., 2017), we assumed that a specific TSA concentration was required for the de-repression of the genes that control the establishment of the root pole in the embryo-like structures. Although this assumption needs experimental verification, TSA and other HDAC inhibitors were demonstrated to affect the expression of genes in a culture of human cells in a concentration-dependent manner (Xu et al., 2007).

A high TSA concentration of 50.0 μM was demonstrated to be required to induce an SE response in the postgermination tissue of Arabidopsis (Tanaka et al., 2008). In contrast, we demonstrated that 0.1 μM of TSA is sufficient to trigger an embryonic response in a culture of IZE explants and that the differences between the TSA concentrations that promote SE in seeds vs. IZEs might reflect different histone acetylation-related repressive states in the postgerminative vs. embryonic tissue.

### A TSA-Promoted SE Response Is Associated With Auxin Biosynthesis and ROS Production

We found that the TSA-induced SE was associated with a high and transient increase of the auxin content in the explants. An IAA surge has also been observed during SE that induced in various plants using different factors (Michalczuk et al., 1992; Charrière et al., 1999; Pasternak et al., 2002; Wójcikowska et al., 2013; Grzyb et al., 2017; Wójcik et al., 2017). Thus, TSA seems to trigger an embryogenic response *via* a common embryogenic pathway that involves stimulating auxin production in the somatic tissue. In support of this, we found that the *YUC1* and *YUC10* encoding the YUC enzymes of the central IAA biosynthesis pathway in Arabidopsis (Kasahara, 2016) were significantly up-regulated in the TSA-treated culture. In addition, we found that both of these genes were expressed on the adaxial side of the cotyledons, which are the explant areas that are involved in the embryogenic response. Relevant to TSA-induced SE, the function of *YUC1* and *YUC10* has also been observed during zygotic embryogenesis (Cheng et al., 2007) and in SE that was induced as a result of *LEC2* overexpression and a *phytoglobin 2* (*pgb2*) mutation (Wójcikowska et al., 2013; Godee et al., 2017). In addition, it was suggested that *YUC1* marks the future somatic embryo initiation sites in the Arabidopsis callus (Bai et al., 2013).

Notably, we also observed the expression of *YUC1* and *YUC10* in the hypocotyl, which is not involved in somatic embryo production. Given that non-cell autonomous auxin biosynthesis seems to be of importance in plant development including zygotic embryogenesis reviewed in Robert et al. (2015), the auxin that is produced in the explant hypocotyls might also contribute to SE induction. In support of this assumption, the auxin transporter PIN1 was demonstrated to mediate the preferential accumulation of IAA in the SE-responsive cells of IZE-cotyledons (Elhiti et al., 2013). The impact of the non-cell autonomous auxin biosynthesis on SE induction should be addressed in future studies.

The intensive crosstalk between ROS and auxin has been reported to control the cell responsiveness to different developmental stimuli (Tognetti et al., 2017). Accordingly, auxin-induced embryogenic cultures were observed to accumulate ROS (Kairong et al., 1999; Elhiti and Stasolla, 2015; Cheng et al., 2015, 2016), and similarly, we found the intensive production of superoxide anion (O_2_^.-^) in the TSA-treated explants. It is noteworthy that ROS accumulation was not observed in the TSA-treated roots of *Populus* (Ma et al., 2016) and this finding strengthens the assumption that ROS are produced in relation to the auxin accumulation rather than in response to the TSA treatment *per se*. The role of ROS in plant development and stress responses has been widely demonstrated reviewed in Choudhury et al. (2017) and Mittler (2017) and among the different free radicals, nitrate oxide (NO) has been found to control auxin biosynthesis during SE induction through the inhibition of the *MYC2* repressor of IAA biosynthesis (Elhiti et al., 2013; Godee et al., 2017). Moreover, a link between NO and histone acetylation-mediated regulation of gene expression in stress response has recently been demonstrated (Mengel et al., 2017). Thus, the ROS that are commonly produced during both auxin- and TSA-induced SE might provide a functional signal in the de-repression of the SE-regulatory genes including those that are involved in auxin biosynthesis.

Taken together, these results suggest that the mechanism of TSA-induced SE is associated with the *YUC1* and *YUC10*-mediated auxin biosynthesis and as a result of auxin and ROS accumulation, the embryogenic transition is triggered in the embryogenically competent explant tissue of the adaxial side of cotyledons.

### TSA-Treatment Results in the Intensive Up-Regulation of the SE-Involved TF Genes

In order to reveal the genetic components of the TSA-triggered embryogenic pathway, we profiled the expression of several genes encoding TFs for which the role in SE induction was documented including *LEC1*, *LEC2*, *FUSCA 3* (*FUS3*), *AGL15*, *BBM*, *EMK/CHO1/AIL5/PLT5*, *WUS*, and *MYB118* (Lotan et al., 1998; Stone et al., 2001; Boutilier et al., 2002; Zuo et al., 2002; Harding et al., 2003; Gaj et al., 2005; Wang et al., 2009; Tsuwamoto et al., 2010). We found that TSA treatment substantially up-regulated the analyzed *TF*s except for *WUS*. The TSA-induced up-regulation of the *LECs* and the *LEC1-type HAP3* was also reported in Arabidopsis seedlings and the IZEs of *Picea abies*, respectively (Tanaka et al., 2008; Uddenberg et al., 2011). Similar to plants, TSA was found to de-repress the transcription of the *TF* genes that control the reprogramming of mammal somatic cells into embryonic stem cells (Hattori et al., 2004).

The majority of SE-regulators exert their function in SE induction *via* the control of the hormone-related pathways including LEC1, LEC2, and FUS3 reviewed by Boulard et al. (2017) and Jia et al. (2014), AGL15 (Zheng et al., 2016), BBM (Horstman et al., 2017b) and EMK (Yamagishi et al., 2008; Yano et al., 2009). Some of these TFs might contribute to the *YUC*-mediated auxin biosynthesis in the TSA-induced explants and accordingly, *LEC1* directly activated the expression of *YUC10* in seedlings (Junker et al., 2012) and *LEC2* stimulated *YUC1* and *YUC10* during SE induction (Wójcikowska et al., 2013). Moreover, *BBM* might indirectly impact the *YUC*-mediated auxin biosynthesis *via* the activation of *LEC1* and *LEC2* in the SE-induced explants (Horstman et al., 2017b).

In auxin-induced SE, *LEC2* expression is activated by the *PHB* gene that is under the repressive control of miR165/166 (Wójcik et al., 2017). Consistent with this, we found *LEC2* up-regulation to be associated with an increased *PHB* expression and the repression of miR166 in the TSA-induced culture (**Supplementary Figure S6**). This result provides additional evidence that there is some convergence of the TSA- and 2,4-D-induced embryogenic pathways.

Besides auxin, stress factors are postulated to play pivotal roles in triggering the embryogenic response, and accordingly, numerous stress-related *TF* genes were differentially expressed during SE that was induced in Arabidopsis on the auxin medium (Gliwicka et al., 2013; reviewed in Nowak and Gaj, 2016). Similarly, a stress-response might also contribute to TSA-triggered SE given that we found that *MYB118*, which has stress-related functions (Zhang et al., 2015), was highly up-regulated in the TSA-induced culture. *MYB118* controls seed maturation *via* the regulation of the biosynthesis of storage compounds (Barthole et al., 2014) and the targets of *MYB118* involve numerous genes of the *LATE EMBRYOGENESIS ABUNDANT* (*LEA*) group (Zhang et al., 2009). We found three of these genes including *EM1*, *EM6*, and *EM10* encoding the proteins that are rich in hydrophilic amino acids and that have a high hydrophilicity and heat stability to be up-regulated in the TSA-induced culture (**Supplementary Figure S7**). Relevantly, seed maturation products were postulated as enhancing the embryogenic competence of IZEs that are treated with 2,4-D possibly by providing a stress-protective environment (Stone et al., 2008; Gliwicka et al., 2012). In conclusion, as in auxin-induced SE, TSA-induced embryogenesis seems to be associated with the activation of the stress-related responses and in support of this, the essential role of HDACs in the regulation of gene expression in the plant response to environmental stress has been demonstrated (Luo et al., 2017).

Despite a general similarity between the TSA- and 2,4-D-induced patterns of the expression of *TF*s, the gene transcript level was distinctly higher in the TSA-induced culture (**Supplementary Figure S5**). Thus, the potential of TSA for gene de-repression seems to be stronger than that of 2,4-D and in line with this assumption, only TSA is capable of activating the SE-regulatory genes in the postgermination tissue of Arabidopsis (Tanaka et al., 2008).

Although the role of *WUS*, which is a member of the *WOX* gene family, in SE induction was reported (Zuo et al., 2002; Su et al., 2009), we did not detect any *WUS* transcripts in the TSA-induced explants. Recently, a very low level of *WUS* transcripts was shown in embryogenic cells isolated from the auxin-induced IZE explants of Arabidopsis (Magnani et al., 2017), thus implying technical limitations in the RT-qPCR detection of *WUS* transcripts in a mixed population of embryogenic and non-embryogenic explant cells. Therefore, spatiotemporal analysis using a relevent reporter line would be of interest in order to gain insight into the *WUS* expression in the TSA-induced explants.

### Histone Acetylation Preferentially Activates the Expression of the Auxin-Responsive SE-Involved TF Genes

A global gene expression analysis indicated a non-stochastic induction of genes via TSA, and accordingly, only a small subset of genes respond to TSA treatment in human and Arabidopsis (Chang and Pikaard, 2005; reviewed in Xu et al., 2007). The results of the present study imply that the SE-involved *TF* genes seem to be among the targets of TSA. Similarly, the *TF* genes that have a central function in the zygotic genome activation were preferentially stimulated in TSA-treated mouse somatic cells (Inoue et al., 2015).

In line with reports demonstrating the TSA-induced inhibition of HDACs (Tai et al., 2005; Ma et al., 2016; Mengel et al., 2017), we observed that the activity of HDACs was the lowest in the TSA-induced explants of Arabidopsis. Thus, a decreased level of histone deacetylation might contribute to the up-regulation of the SE-involved *TF* genes in the TSA-treated explants. In support of this, the involvement of the histone deacetylase complex was implicated in the repression of the genes of the *LAFL* network that involves the *LEC2* and *FUS3* genes (Jia et al., 2014) with a TSA-stimulated expression (present results).

The significant differences (up to 255-fold) in the TSA-induced transcription level that were observed between the *TF* genes suggest that distinct HDACs that have individual sensitivities to TSA might specifically regulate the promoters of the *TF* genes (Weiste and Dröge-Laser, 2014). However, it is also possible that TSA indirectly affects the expression of the SE-involved *TF* genes and therefore further studies are needed to evaluate the histone acetylation level in the promoters of the *TF*s in response to TSA.

In order to regulate gene expression, HDACs cooperate with HATs and the complex physical and functional interplay between these enzymes was demonstrated to control cell acetylome (reviewed in Pfluger and Wagner, 2007; Peserico and Simone, 2011) In line with this belief, we found that the decreased activity of HDACs was associated with a reduced activity of HATs. Notably, the explants cultured on different media displayed a reduced acitvity of deacetylases and acetylases, thereby suggesting that *in vitro* culture conditions *per se* might account for this result. Although, to the best of our knowledge, data on the acetylase/deacetylase activity during an *in vitro* culture are not available, the modulated expression of the specific *HDAC* and *HAT* genes in response to stress (reviewed in Chinnusamy and Zhu, 2009), which is inevitably associated with the explant culture together with reports on somaclonal epigenetic variants that have a deregulated deacetylase activity (Yaacob et al., 2013; Halim et al., 2017) provide indirect support on the possible impact of *in vitro* culture conditions on the (de)acetylase activity.

The current model of auxin-regulated gene expression implies a relationship between the auxin- and TSA-responsiveness of genes reviewed in Weijers and Wagner (2016). In line with this model, we identified AuxREs (Auxin Response Elements) in the promoters of the majority (five out of seven) *TF* genes that have a TSA-up-regulated expression (**Supplementary Table S2**). Similarly, the majority (60%) of the *TF* genes that were differentially expressed in auxin-induced SE of Arabidopsis had the AuxREs in their promoters (Gliwicka et al., 2013; AGRIS^3^). Among the candidate targets of TSA, numerous *ARFs* (*AUXIN RESPONSE FACTORS*) that have an auxin-regulated expression in SE might be considered (Gliwicka et al., 2013; Wójcikowska and Gaj, 2017). Consistent with this, we observed a down-regulation of *ARF10* and *ARF17* in the TSA-induced explants (**Supplementary Figure S8**). Similarly, TSA was demonstrated to down-regulate numerous genes that are involved in auxin signaling in Arabidopsis (Chang and Pikaard, 2005) and *Populus* (Ma et al., 2016). Notably, in contrast to the TSA-induced down-regulation of *ARF10* and *ARF17*, auxin-treatment resulted in an increased expression of these genes (Wójcikowska and Gaj, 2017). Thus, although TSA seems to preferentially target the auxin-responsive genes, the effects of TSA vs. 2,4-D on the gene expression level might differ.

Interestingly, we found that treatment of the explants with TSA in combination with 2,4-D and other auxins (IAA and NAA) resulted in the inhibition of the embryogenic response possibly due to the repression of the *LEC1*, *LEC2*, and *BBM* genes that control the auxin biosynthesis that is associated with SE induction (Wójcikowska et al., 2013; Horstman et al., 2017b). Given that SE induction requires auxin biosynthesis, we assumed that the inhibition of the positive regulators of auxin biosynthesis prevents auxin accumulation and SE induction on the medium supplemented with TSA and auxin. In addition to auxin biosynthesis, auxin transport, which plays a key role in the embryogenic response that is induced *in vitro* (Chen and Chang, 2004; Cueva-Agila et al., 2016), might be also affected in explants that are treated with TSA and auxin. In support of this hypothesis, the Arabidopsis seedlings treated with the HDAC-inhibitor and auxin exhibited a defective development of roots, which was attributed to the degradation of the PIN1 protein (Nguyen et al., 2013). Further studies are required in order to reveal the auxin-related epigenetic mechanism that underlies the contrasting effects induced in the explants treated with TSA alone and in combination with auxins.

Importantly, in addition to histones, non-histone proteins were also observed among the HDAC-targets that involve the proteins of the transcriptional complexes (Spange et al., 2009; Duffy et al., 2012) and other polypeptides such as hormone receptors, chaperones and cytoskeleton proteins, which regulate cell proliferation and cell death (Xu et al., 2007; Hartl et al., 2017). Thus, the TSA-induced SE mechanism might also comprise unrelated modifications to gene transcription that at present remain unexplored. Moreover, TSA also affects other epigenetic processes including the methylation of DNA and histones (Kishigami et al., 2006; Ou et al., 2007; Yang et al., 2010). Hence, understanding the intricate mechanism that govern the de-repression of the embryogenic potential in somatic plant cells requires deciphering the complex regulatory network in which histone and non-histone acetylation and various epigenetic processes interplay to regulate gene expression.

## Author Contributions

MG and BW conceived and designed the experiments, analyzed the data, and wrote the manuscript. MB, JM, BW, and AW performed the experiments. TN and JK contributed to the imaging with the confocal laser scanning microscope and SEM, respectively. All of the authors read and approved the manuscript.

## Conflict of Interest Statement

The authors declare that the research was conducted in the absence of any commercial or financial relationships that could be construed as a potential conflict of interest.

## Footnotes

1 ^1^http://www.epigentek.com/catalog/epigenase-hdac-activityinhibition-direct-assay-kit-colorimetric-p-2867.html

2 ^2^https://www.epigentek.com/catalog/epiquik-hat-activityinhibition-assay-kit-p-951.html

3 ^3^http://arabidopsis.med.ohio-state.edu/AtcisDB/

## Supplementary Material

FIGURE S1 Enhanced accumulation of neutral lipids and ROS suggest an embryogenic character of the TSA-induced morphogenic pathway. The IZE explants of Col-0 were induced toward alternative developmental processes including seedling development (E0), shoot organogenesis (CIM/SIM) and SE (ET and EA). Neutral lipids **(A)** and superoxide anion O_2_^.-^ **(B)** were stained with Sudan Red 7B and NBT, respectively.

FIGURE S2 The TSA-induced embryogenic response was reduced on the media supplemented with IAA and NAA. The IZE explants of Col-0 were cultured on ET medium with IAA and NAA at different concentrations and the SE efficiency and productivity was assessed in relation to the ET medium that was set at 100% (*n* = 3; means ± SE).

FIGURE S3 RT-PCR analysis of the *YUC* gene expression in the cultures of the IZE, Col-0 explants induced on the E0 and ET (E0 + 1.0 μM TSA) media for 5, 10, 15, 21, and 28 days. The *TIN* gene (*At4g27090*) was used as the control for the cDNA synthesis.

FIGURE S4 Transcripts of *WUS* were not detected in IZE Col-0 explants cultured on E0 and ET (E0 + 1.0 μM TSA) media for 0, 5, 10, 15, 21, and 28 days analyzed with RT-PCR. The *At4g27090* gene a was used as the control for the cDNA synthesis. NC, negative control.

FIGURE S5 RT-qPCR analyzed the expression level of the *MYB118* **(A)**, *LEC1* **(B)**, *LEC2* **(C)**, *AGL15* **(D)**, *FUS3* **(E)**, *BBM* **(F)**, and *EMK* **(G)** genes in the TSA-induced (1.0 μM of TSA) culture in relation to the gene expression level in the 2,4-D-induced (E5) culture (Grzybkowska et al., 2018). The IZE explants of Col-0 were cultured for 5, 10, and 15 days and sampled for RT-qPCR analyses. Their relative transcript level was normalized to the internal control (*At4g27090*) and calibrated to the culture on E5. Values that were significantly different to those observed on E5 at the same age of culture are indicated by an asterisk (^∗^); (*n* = 3; means ± SE; the Student’s *t*-test *P* < 0.05).

FIGURE S6 Relative amount of mature miR166 molecules and transcript level of *PHB* and *PHV* genes during TSA-induced SE. IZE explants of Col-0 were cultured on an ET (E0 + 1.0 μM of TSA) medium and the tissue for analyses was sampled on day 5, 10, 15, 21, and 28 of the culture. Relative transcript level was normalized to the internal control (*At4g27090*) and calibrated to the culture on E0 (*n* = 3).

FIGURE S7 TSA treatment results in up-regulated expression of *EM1* **(A)**, *EM6* **(B)**, and *EM10* **(C)** genes, the targets of MYB118. IZE explants of Col-0 were cultured on an ET (E0 + 1.0 μM of TSA) medium and the tissue for RT-qPCR analyses was sampled on days 5, 10, 15, 21, and 28 of the culture. Relative transcript level was normalized to the internal control (*At4g27090*) and calibrated to the culture on E0 (*n* = 3). Values significantly different to that observed on E0 at the same age of culture are indicated by an asterisk (^∗^); (*n* = 3; means ± SE; the Student’s *t*-test *P* < 0.05).

FIGURE S8 Expression level of the *ARF10*, *ARF16*, and *ARF17* genes during the SE process that was induced by TSA. The IZE explants of Col-0 were cultured on an ET (E0 + 1.0 μM of TSA) medium and the tissue for the RT-qPCR analyses was sampled on days 5, 10, 15, 21, and 28 of the culture. The relative transcript level was normalized to the internal control (*At4g27090*) and calibrated to the culture on E0 (*n* = 3).

TABLE S1 The primers that were used in experiments.

TABLE S2 The auxin-related *cis*-elements localized in the promoter region of the analyzed genes.

## Abbreviations

2,4-D: 2,4-dichlorophenoxyacetic acid
CIM: callus induction medium
Ct: threshold cycle
E0: control medium, free of plant growth regulators
EA: embryogenesis induction the auxin medium (E0 + 2,4-D)
ET: embryogenesis induction the medium with TSA (E0 + TSA)
IZE: immature zygotic embryo
PRC2: polycomb repressive complex 2
SE: somatic embryogenesis
SIM: shoot induction medium
TF: transcription factor
TSA: Trichostatin A
