# Supplementary figures and images for: Trichostatin A Triggers an Embryogenic Transition in Arabidopsis Explants via an Auxin-Related Pathway
Source: Front Plant Sci. 2018 Sep 13;9:1353. doi: 10.3389/fpls.2018.01353 (PMC6146766; doi:10.3389/fpls.2018.01353)

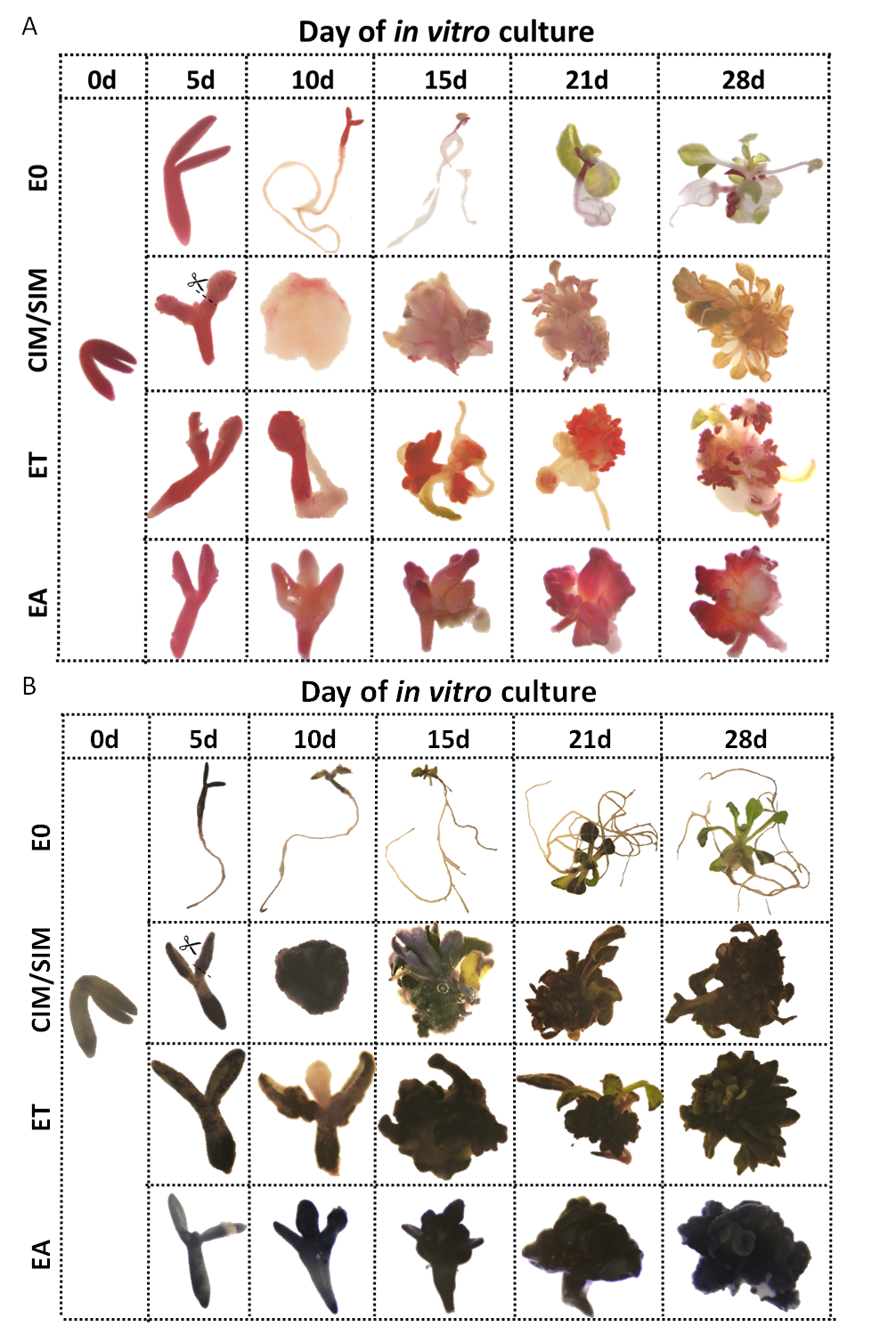

Supplement: FIGURE S1 — Enhanced accumulation of neutral lipids and ROS suggest an embryogenic character of the TSA-induced morphogenic pathway. The IZE explants of Col-0 were induced toward alternative developmental processes including seedling development (E0), shoot organogenesis (CIM/SIM) and SE (ET and EA). Neutral lipids (A) and superoxide anion O2.- (B) were stained with Sudan Red 7B and NBT, respectively. [file Image_1.TIF]

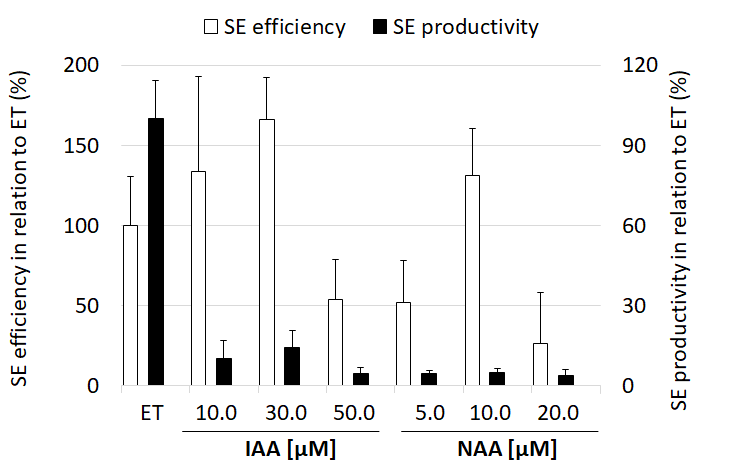

Supplement: FIGURE S2 — The TSA-induced embryogenic response was reduced on the media supplemented with IAA and NAA. The IZE explants of Col-0 were cultured on ET medium with IAA and NAA at different concentrations and the SE efficiency and productivity was assessed in relation to the ET medium that was set at 100% (n = 3; means ± SE). [file Image_2.tif]

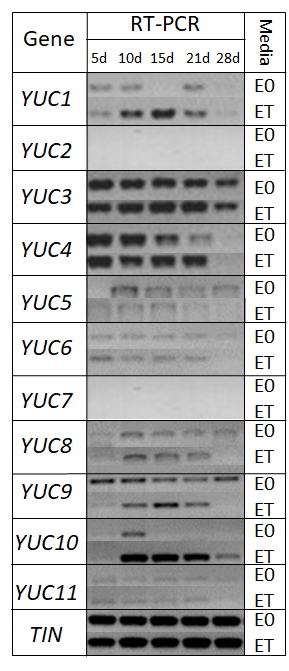

Supplement: FIGURE S3 — RT-PCR analysis of the YUC gene expression in the cultures of the IZE, Col-0 explants induced on the E0 and ET (E0 + 1.0 μM TSA) media for 5, 10, 15, 21, and 28 days. The TIN gene (At4g27090) was used as the control for the cDNA synthesis. [file Image_3.TIF]

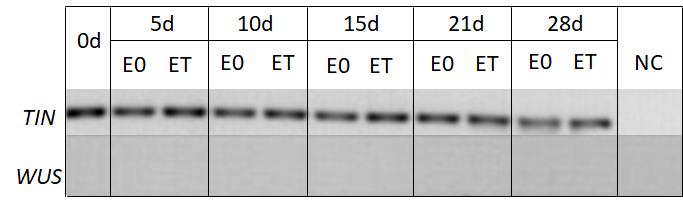

Supplement: FIGURE S4 — Transcripts of WUS were not detected in IZE Col-0 explants cultured on E0 and ET (E0 + 1.0 μM TSA) media for 0, 5, 10, 15, 21, and 28 days analyzed with RT-PCR. The At4g27090 gene a was used as the control for the cDNA synthesis. NC, negative control. [file Image_4.TIF]

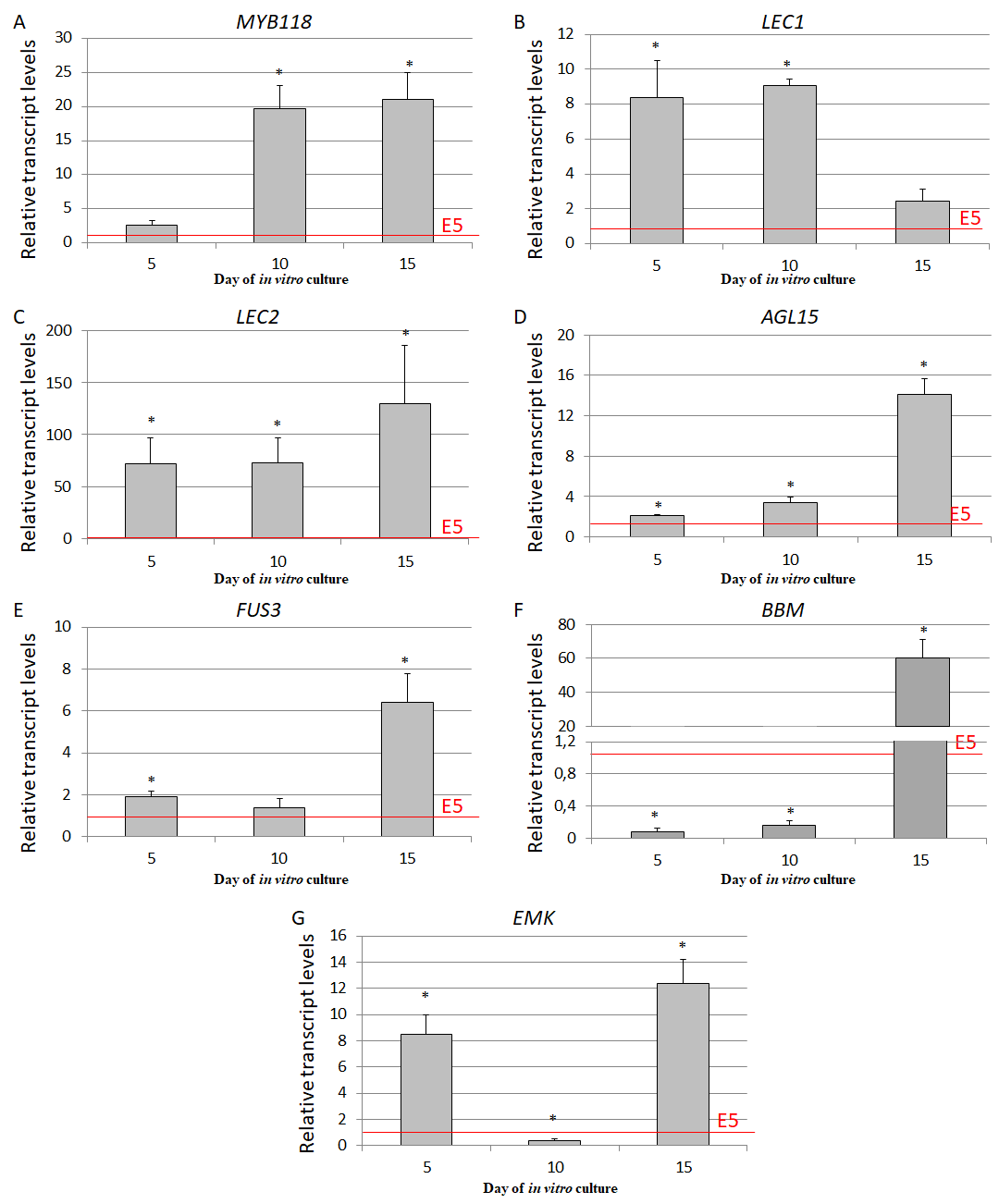

Supplement: FIGURE S5 — RT-qPCR analyzed the expression level of the MYB118 (A), LEC1 (B), LEC2 (C), AGL15 (D), FUS3 (E), BBM (F), and EMK (G) genes in the TSA-induced (1.0 μM of TSA) culture in relation to the gene expression level in the 2,4-D-induced (E5) culture (Grzybkowska et al., 2018). The IZE explants of Col-0 were cultured for 5, 10, and 15 days and sampled for RT-qPCR analyses. Their relative transcript level was normalized to the internal control (At4g27090) and calibrated to the culture on E5. Values that were significantly different to those observed on E5 at the same age of culture are indicated by an asterisk (∗); (n = 3; means ± SE; the Student’s t-test P < 0.05). [file Image_5.tif]

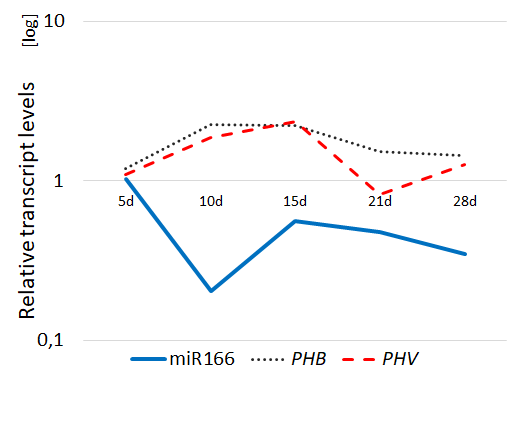

Supplement: FIGURE S6 — Relative amount of mature miR166 molecules and transcript level of PHB and PHV genes during TSA-induced SE. IZE explants of Col-0 were cultured on an ET (E0 + 1.0 μM of TSA) medium and the tissue for analyses was sampled on day 5, 10, 15, 21, and 28 of the culture. Relative transcript level was normalized to the internal control (At4g27090) and calibrated to the culture on E0 (n = 3). [file Image_6.TIF]

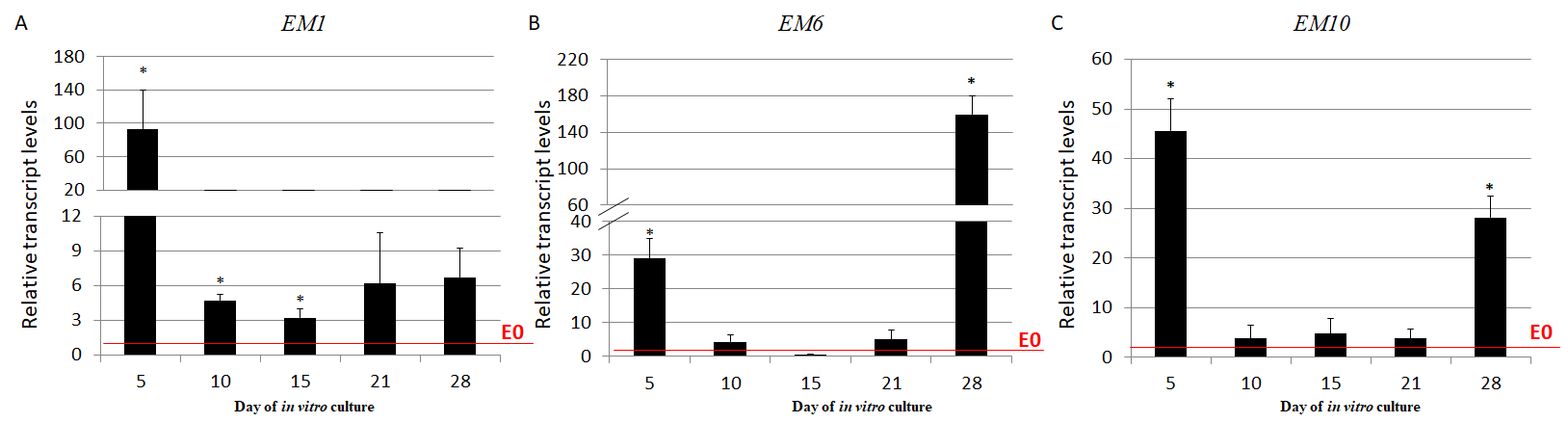

Supplement: FIGURE S7 — TSA treatment results in up-regulated expression of EM1 (A), EM6 (B), and EM10 (C) genes, the targets of MYB118. IZE explants of Col-0 were cultured on an ET (E0 + 1.0 μM of TSA) medium and the tissue for RT-qPCR analyses was sampled on days 5, 10, 15, 21, and 28 of the culture. Relative transcript level was normalized to the internal control (At4g27090) and calibrated to the culture on E0 (n = 3). Values significantly different to that observed on E0 at the same age of culture are indicated by an asterisk (∗); (n = 3; means ± SE; the Student’s t-test P < 0.05). [file Image_7.TIF]

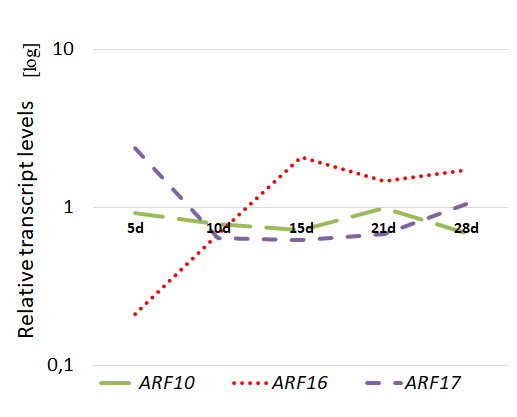

Supplement: FIGURE S8 — Expression level of the ARF10, ARF16, and ARF17 genes during the SE process that was induced by TSA. The IZE explants of Col-0 were cultured on an ET (E0 + 1.0 μM of TSA) medium and the tissue for the RT-qPCR analyses was sampled on days 5, 10, 15, 21, and 28 of the culture. The relative transcript level was normalized to the internal control (At4g27090) and calibrated to the culture on E0 (n = 3). [file Image_8.TIF]
