# Supplementary material for: Trichostatin A Triggers an Embryogenic Transition in Arabidopsis Explants via an Auxin-Related Pathway
Source: Front Plant Sci. 2018 Sep 13;9:1353. doi: 10.3389/fpls.2018.01353 (PMC6146766; doi:10.3389/fpls.2018.01353)
Supplement: TABLE S1 — The primers that were used in experiments. [file Table_1.docx]

**Supplementary Table S1.** **The primers that were used in experiments.**

| **The primers that were used in the RT-qPCR/PCR analyses of the *TF* and *YUC* genes** | | | |
| --- | --- | --- | --- |
| **Gene** | **ID** | **pR 5’-3’** | **pF 5’-3’** |
| *LEC1* | *At1g21970* | CTGGACCACGATACCATTGTT | GTGGAGCTCCCTTCTCTCACT |
| *LEC2* | *At1g28300* | CAGTGGTGAGGTCCATGAGAT | AGGGAAAGGAACCACTACGAA |
| *FUS3* | *At3g26790* | TGAAGGTCCAAACGTGAAAAC | GTCAGCTCTCTCCGACGTATG |
| *PHB* | *At2g34710* | CAGATGAGCATAGCCCTGTTGC | AAGACCCTTGACGAACCTGGTC |
| *PHV* | *At1g30490* | CGGAAGATTCGCATATCCCTGCTG | ACTTGATGACTCTGGTCGTAGAGC |
| *BBM* | *At5g17430* | ATCTACCTGTCCACCGATGC | AATGCTAATCATCAAGACAAT |
| *EMK* | *At5g57390* | AGAGCTGCAAGGTCGTAAGC | TCGCCTACTCCGAAGAAGAA |
| *MYB118* | *At3g27785* | CGAGCTATCTCAGCCCATCT | GGTGCAGCTAGTGGACCTTC |
| *AGL15* | *At5g13790* | GTTGTTCCTTGAGGCGTGAT | CAAGGGCTTGAATCCTCTGA |
| *WUS* | *At2g17950* | AGAACAGTCTTGTTCCATAGA | TCACCATCATCACGGTGTTC |
| *YUC1* | *At4g32540* | CCGGTGACATTTTTCAGCTC | CGGAACACCGTTCATGTGT |
| *YUC2* | *At4g13260* | TTCAAGAGGGCCAAGTTTTG | TTGTGGTTCGTGACTCGGTA |
| *YUC3* | *At1g04610* | TCATGAGCCACACTCATAGC | GATGGCCGTGTTCTTGAGAT |
| *YUC4* | *At5g11320* | AAAAACTATTCTCCTTAAGCCAATC | AACTCCCGTTCTTGATGTCG |
| *YUC5* | *At5g43890* | TTCTCGCCGGATTTGTACTC | TGTCCAGTCTGCTCGATACG |
| *YUC6* | *At5g25620* | TTGGAAATCCATCTTTCTTACTAAAC | GGTAAAACTCCGGTTCTCGAC |
| *YUC7* | *At2g33230* | CCAAGTCGTTTTCCTTAAGCC | TGAAGAACACCGCAGGTAAA |
| *YUC8* | *At4g28720* | AGCCACTGGTCTCATCGAAC | CGTCTCAAGCTTCACCTTCC |
| *YUC9* | *At1g04180* | CGGCGTCTTTCCTGTCAT | TGGTCGTTAGAAGCTCGGTT |
| *YUC10* | *At1g48910* | TCACGTATTCATAGTCCTCTAACCA | TTACCGGAAAAGCTCCTGTC |
| *YUC11* | *At1g21430* | TAACACGTGCACCTGGCTAC | GAGAATGGCGAAGGTGTGAT |
| *TIN* | *At4g27090* | CCTCGATCAAAGCCTTCTTCT | GTCGTTATCGTCGACGTTGTT |
|  | | | |
| **The primers that were used in the miR166 analysis** | | | |
| RT-qPCR | GTGCAGGGTCCGAGGT (pR 5’-3’) | | TCGCGTGAAGCTGCCAGCAT (pF 5’-3’) |
| *Stem-loop RT 5’-3’* | GTTGGCTCTGGTGCAGGGTCCGAGGTATTCGCACCAGAGCCAACGGGGAA | | |
