# Supplementary material for: Trichostatin A Triggers an Embryogenic Transition in Arabidopsis Explants via an Auxin-Related Pathway
Source: Front Plant Sci. 2018 Sep 13;9:1353. doi: 10.3389/fpls.2018.01353 (PMC6146766; doi:10.3389/fpls.2018.01353)
Supplement: TABLE S2 — The auxin-related cis-elements localized in the promoter region of the analyzed genes. [file Table_2.docx]

**Supplementary Table S2. The auxin-related cis-elements localised in the promoter region of the analysed** **genes.**

| **Gene** | **ID** | **AuxRE** | **G-box** |
| --- | --- | --- | --- |
| *LEC1* | At1g21970 | **+** | - |
| *LEC2* | At1g28300 | **+** | - |
| *FUS3* | At3g26790 | **+** | - |
| *BBM* | At5g17430 | **+** | - |
| *WUS* | At2g17950 | **+** | - |
| *EMK* | At5g57390 | **+** | **+** |
| *MYB118* | At3g27785 | - | - |
| *AGL15* | At5g13790 | - | - |
